# Biological and antibacterial properties of a new silver fiber post: In vitro evaluation

**DOI:** 10.4317/jced.53464

**Published:** 2017-03-01

**Authors:** Claudio Poggio, Federico Trovati, Matteo Ceci, Marco Chiesa, Marco Colombo, Giampiero Pietrocola

**Affiliations:** 1 MD, DDS, Department of Clinical-Surgical, Diagnostic and Pediatric Sciences, Section of Dentistry, University of Pavia, Pavia, Italy; 2 DMD, Department of Clinical-Surgical, Diagnostic and Pediatric Sciences, Section of Dentistry, University of Pavia, Pavia, Italy; 3 DMD, PhD , Department of Clinical-Surgical, Diagnostic and Pediatric Sciences, Section of Dentistry, University of Pavia, Pavia, Italy; 4 MD, DDS, Department of Molecular Medicine, Unit of Biochemistry, University of Pavia, Pavia, Italy

## Abstract

**Background:**

The incorporation of nano silver particles (AgNPs) to improve antibacterial properties of dental materials has become increasingly common. The aim of the present study was to compare the antibacterial activity and cytotoxicity effects of different fiber posts: glass fiber post, quartz fiber post, nano fiber post and silver fiber post.

**Material and Methods:**

The antibacterial activity against *S. mutans*, *S. salivarius* and *S. sanguis* was evaluated by using the agar disc diffusion test (ADT). Four wells of 3x2 mm (one for each material) were made with a punch by removing the agar and filled with the materials to be evaluated. The size of the inhibition zone was calculated. An extract was made eluting the posts in cell culture medium using the surface area-to-volume ratio of approximately 1.25cm²/ml between the surface of the samples and the volume of medium. Cell cultures were then exposed to 100 μL of the extracts medium. After 24 h, cell viability was determined using the MTT assay.

**Results:**

Silver fiber post was the only material showing a fair antibacterial effect against all the three streptococcal strains. The level of cytotoxicity of all the fiber posts tested was higher than 90% and therefore they were considered not cytotoxic.

**Conclusions:**

The new silver fiber post reported a fair antibacterial activity. On the other hand all the fiber posts tested (including the post with incorporated AgNPs) proved to be biocompatible, suggesting that their application does not represent a threat to human health.

** Key words:**Antibacterial activity, agar disc diffusion test, biocompatibility, fiber post, MTT test.

## Introduction

Fiber posts are generally needed in endodontically treated teeth with great coronal destruction to obtain better retention of crowns or resin composite restorations. Although there is no evidence that endodontically treated teeth are reinforced by posts, it is recognized that fiber posts better distribute the stress generated on teeth under function (1,2).

The ultimate goal of endodontic treatment is the eradication of microorganism from the root canal space, or at least their reduction to levels compatible with periradicular tissue health (3). Although, after chemo mechanical treatment of root canals the population of microorganisms is significantly decreased, all the microorganisms cannot be eliminated (4). Many studies reported the presence of bacteria in dentinal tubules and cementum after endodontic treatment (5).

Therefore, endodontic sealers with high antimicrobial activity helps to decrease or prevent the growth of microorganisms and aid the repair process of apical and periapical tissues (6). For the same reasons, some recent studies have evaluated the antibacterial activity of resin composites for intracanal post cementations and core build-up restorations (7,8).

Metallic silver is an element commonly used in dentistry. Silver has many advantages, such as low toxicity and good biocompatibility with human cells, long-term antibacterial activity, due to sustained ion release, and low bacterial resistance (9,10). The anti-bacterial activity of silver has been demonstrated to be superior if compared to other metals; silver presents high cytotoxic effects on a broad range of microorganisms in both metallic and ionic forms (11).

With the advent of nanotechnology, silver nanoparticles (AgNPs) have been synthesized. The antibacterial properties of AgNPs on oral pathogens were recently assessed; supporting their use in dental materials (12). AgNPs incorporation aims to avoid or at least to decrease microbial colonization. AgNPs have been applied in several areas of dentistry, such as endodontics (13), dental prostheses (14), implantology (15) and restorative dentistry (16). Therefore various studies have shown that AgNPs addition did not affect the viability of human fibroblasts cells, evidencing the clinical applicability of this antimicrobial (17).

MicroMedica S.r.l. (Robbio, PV, Italy) recently developed a new fiber post with incorporated AgNPs. The purpose of the producers is to create a fiber post which joins to the usual elasticity, mechanical strength, adhesion and aesthetic characteristics, a new antibacterial capacity given by the incorporation of silver.

By using the the agar disc diffusion test (ADT) and the MTT assay, the aim of the present study was to compare the antibacterial activity and the cytotoxicity effects of different fiber posts: glass fiber post, quartz fiber post, nano fiber post and silver fiber post. The null hypothesis of the study was that the fiber posts tested did not demonstrate cytotoxic and antibacterial effects; therefore that there was no difference between the different posts tested.

## Material and Methods

-Post sample preparation

Fiber posts selected for this study: fiber glass post, quartz fiber post, nano fiber post, silver quartz fiber post (silver fiber post). Sterile wells of different fiber posts (diameter: 3 mm, thickness: 2 mm) were made.  Table 1 shows composition of the posts tested.

**Table 1:**
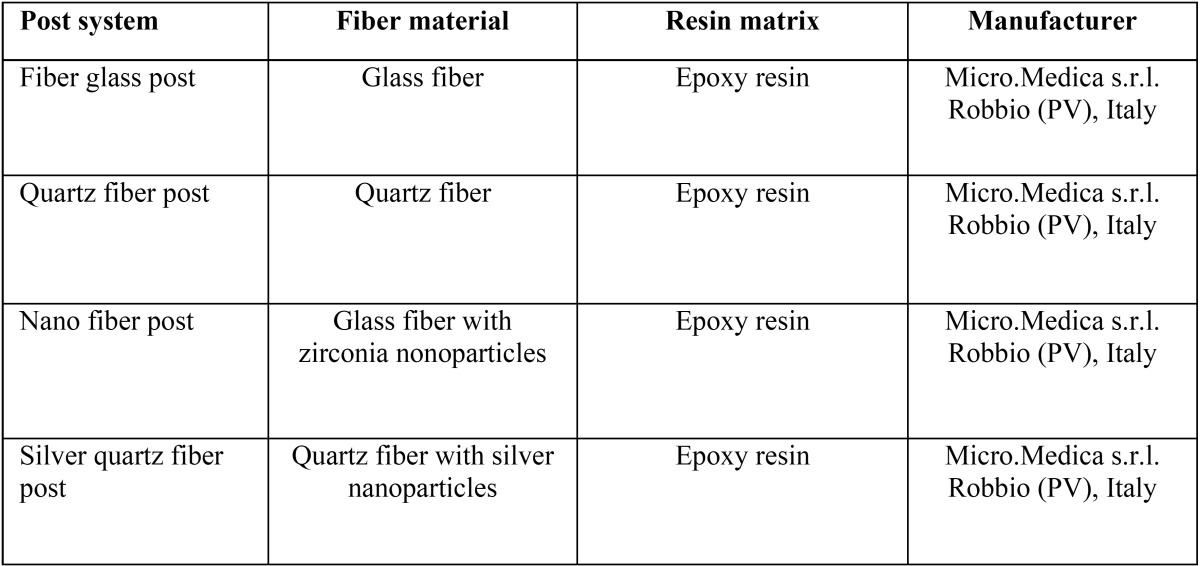
Fiber posts tested in this study.

-Agar diffusion test (ADT)

The microbiological assays were carried out under aseptic conditions in a laminar flow chamber (Foster Wheeler Italiana S.p.A, VBH C2 biohazard cabine). The antibacterial activity was evaluated using standard strains of *Streptococcus mutans* (CCUG 35176), *Streptococcus salivarius* (CCUG 11878) and *Streptococcus sanguis* (CCUG 17826). The microorganisms were cultivated in Brain Heart Infusion (BHI) (Difco Lab., Detroit, MI, USA) supplemented with 5% (v/v) heat-inactivated defibrinated horse blood serum (OxoidS.p.A., Rodano, MI, Italy) at 37°C for 18 h under anaerobic conditions. The overnight culture were then adjusted to the turbidity of 1.0 McFarland standard tube, corresponding to 1,5 x 108 CFU / mL. Six replica plates containing Mueller-Hinton agar (Difco Lab., Detroit, MI, USA), 5% (v/v) heat-inactivated defibrinated horse blood serum (Oxoid S.p.A., Rodano, MI, Italy) and 20 mg/L β-NAD (MHF) (Sigma, St. Louis, MO, USA) were spread with 0.1 mL of the bacterial suspension, using a Drigalsky’s loop. Thereafter, four wells of 3 mm in diameter and 2 mm in depth (one for each material) were made with a punch by removing the agar at equidistant points and then filled immediately with the materials to be evaluated. Two plates did not receive the bacterial suspension; one did not receive the posts and aimed to control the sterilization of the culture medium, whilst the other received the sealers and aimed to control their contamination. All plates were maintained at room temperature for 2 h for prediffusion of the materials and then incubated at 37°C for 48 h under aerobic conditions. The inhibition zones around each one of the wells were then measured by the same operator in two perpendicular locations with a millimeter ruler (sliding calipers) with accuracy of 0.5 mm. The size of the inhibition zone was calculated as follows: Size of inhibition zone = (diameter of halo – diameter of specimen) x ½

All the assays were conducted in triplicate and the results were recorded in terms of the average diameter of inhibition zone ( Table 2).

**Table 2:**
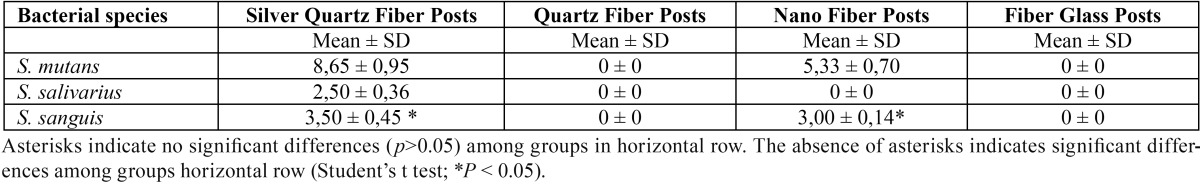
Mean diameter ± standard deviation (mm) of the bacterial inhibition zone by posts materials evaluated after 48h by ADT.

-Cell culture

Human Periodontal Ligament Fibroblasts (HPdLF) (Lonza, Visp, Switzerland) were cultured in high glucose Dulbecco’s modified Eagle’s medium (DMEM; Sigma-Aldrich, St. Louis, MO, USA) supplemented with 4 mM L-glutamine (Sigma-Aldrich), 1% penicillin, streptomycin (Sigma-Aldrich) and 10% (vol/vol) heat-inactivated fetal bovine serum (FBS; Sigma-Aldrich). Cells were incubated at 37°C in 5% CO2 atmosphere, fed every 48 h and routinely sub-cultured every 5 -days with a split ratio of 1:3 using trypsin-EDTA (0.05%; Sigma-Aldrich) for 3 min at 37°C.

-Preparation of the extract

The extraction was made eluting the posts in cell culture medium (see cell culture paragraph) using the surface area-to-volume ratio of approximately 1.25cm²/ml between the surface of the samples and the volume of medium (International Organization for Standardization. ISO 10993-5: Biological evaluation of medical devices, part 5: tests for cytotoxicity: *in vitro* models. 1st edition. Geneva: ISO; 1997). The extraction vials were the incubated at 37°C for 24 hours, 48 hours or 72 hours. The specimens were then discarded and the eluate extracts were filtered by 0.22-μm pore size membranes (Millipore; Billerica, MA, USA). Control samples containing only culture medium were similarly treated. Undiluted extracts were used for the testing.

-Cytotoxicity Test

Cells (1×104) were seeded in each well of a 96-well plate and incubated for 24 h at 37°C. Cultures were then exposed to 100 μL of the extracts medium. Cell cultures with supplemented DMEM (FBS and antibiotics solution) were used as controls. After 24 h, cell viability was determined using the MTT assay. The MTT solution (3-{4,5-dimethylthiazol-2-yl}-2,5-diphenyl tetrazolium bromide) (Sigma-Aldrich) in RPMI-1640 without phenol red (Sigma-Aldrich) (5 mg/mL) was added to each well of culture plate to make final concentration of 0.5 mg/mL and the cells were incubated for 4 h at 37°C. Then, the supernatant was removed and the resulting formazan was dissolved by adding 100 μL DMSO (Sigma-Aldrich) to each well. The optical density of formazan dye was read at 545 nm against 620 nm as background by Elisa reader (Bio-Rad, Hercules, California, USA). The percentage of viable cells in each well was calculated relative to control cells set to 100%. Cytotoxicity responses were rated as severe (30%), moderate (30-60%), mild (60-90%) or non cytotoxic (>90%) (18).

-Statistical analysis

Statistical analysis was carried out using Graph Pad Prism statistical analysis software. Differences between groups were analyzed by ANOVA with the appropriate post-test and by using repeated measures where required. A *p* value < 0.05 was considered statistically significant.

## Results

-Antimicrobial Activity

**Figure 1:**
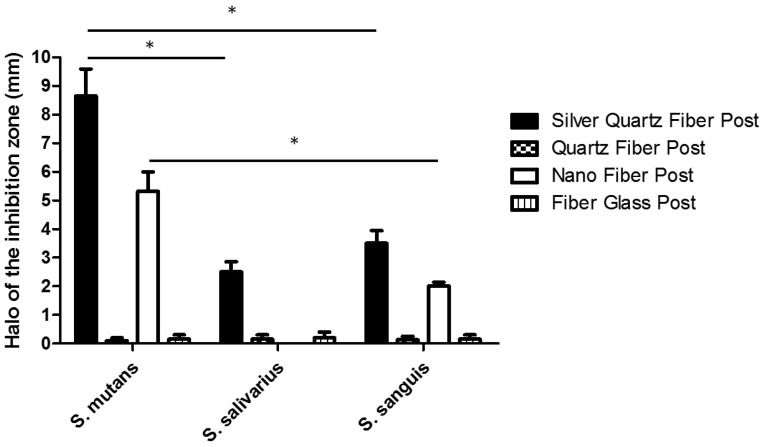
Antibacterial activity of different post materials evaluated by agar diffusion test. Each post was placed on agar plates previously incubated with the indicated streptococcal strains and incubate at 37°C for 48h. All the assays were conducted in triplicate and the results were recorded in terms of the average diameter of inhibition zone (mm). Error bars indicate standard errors of the means. Statistically significant differences are indicated (Student’s t test; *, *P*<0.05).

The antimicrobial activity of the tested fiber post materials was evaluated with the agar diffusion test. As shown in figure 1, silver quartz fiber post was the only material showing a fair antibacterial effect against all the three streptococcal strains. Moreover, significantly higher (*P*<0.05) was the antimicrobial activity on *S. mutans* compare to observed on *S. salivarius* or *S. sanguis*. Its effect is also significantly higher (*P*<0.05) than that observed for all the other fiber posts tested. Nano fiber post was effective on *S. mutans* and significantly (*P*<0.05) lesser extent also on *S. sanguis*. No antimicrobial activity for both quartz fiber post and fiber glass post was detected. There was no bacterial growth on the two control plates.

-Biocompatibility

**Figure 2:**
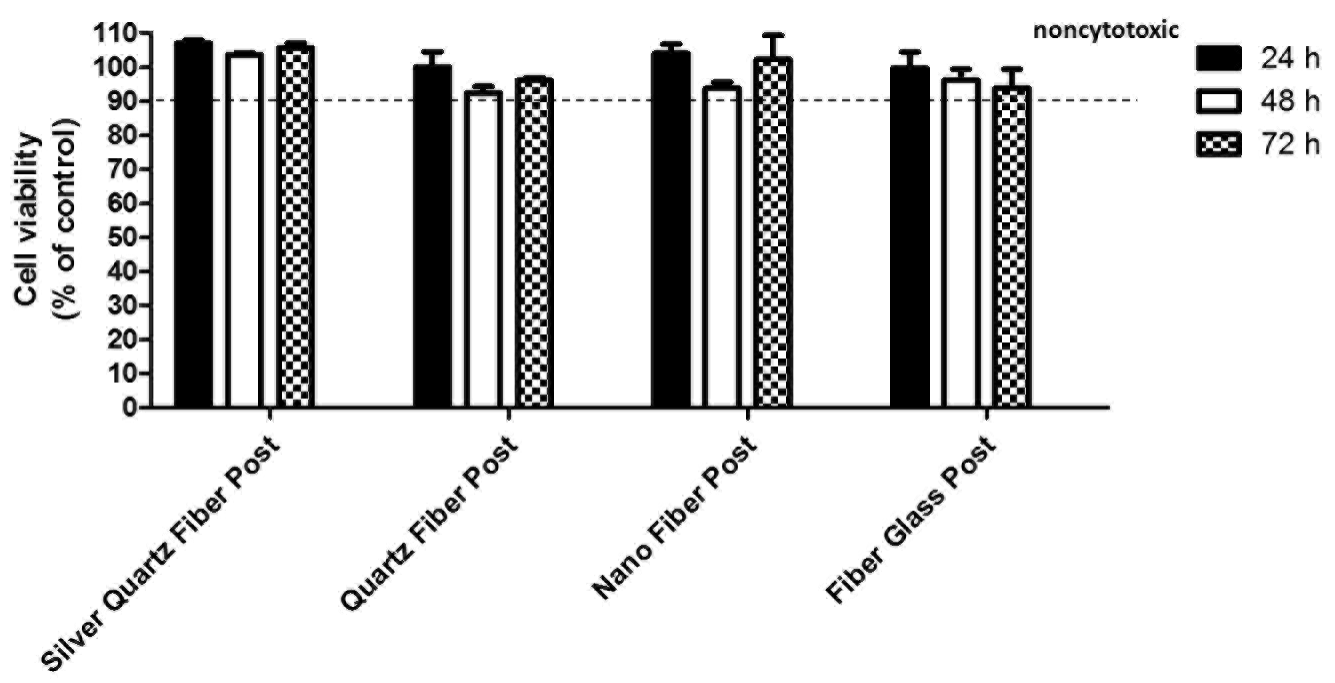
Cell viability after treatment with extracts from four different posts. Confluent human gingival fibroblast were treated for 24 hours with extracted medium made eluting the posts for 24 hours, 48 hours or 72 hours. The cell viability was mesured by the MTT assay. Values are expressed as percentages relative to the control group (100% viability). Bars and error bars represent the means and ± SD from three independent determinations performed in triplicate.

The cell viability of human periodontal fibroblasts in contact with the extracts was evaluated by means of MTT assay. As shown in figure 2, the level of cytotoxicity of all the fiber posts tested was higher than 90% and therefore considered not cytotoxic (18). To support the MTT data, the cell growth and morphology on each material were evaluated by optical microscopy. As expected, well-spread and flattened cells were observed (data not shown).

## Discussion

Although many improvements have been achieved during recent years, the protocols of endodontic chemo-mechanical disinfection used today cannot predictably provide the sterility of root canal complex. As none of the elements of endodontic therapy (host defense system, instrumentation and irrigation, intracanal medicaments, permanent root ﬁlling and coronal restoration) can guarantee complete disinfection (19).

Since complete eradication of microorganism from the endodontic space is not predictable; the antimicrobial activity of root canal sealers may help to eliminate residual microorganisms unaffected by chemo-mechanical preparation of the root canal system (6,20). The incorporation of antimicrobial components into root canal sealers may become an essential factor in preventing the re-growth of residual bacteria (6,21). Similarly, recent studies have demonstrated that the addition of antibacterial agents within polymerizable formulations can provide a novel approach to prepare reinforced resin composite material for intracanal post cementation and core build-up (8).

Fiber posts have been widely evaluated for their aesthetic properties (22), bond strength (23), mechanical characteristics (24) and degradation (25); also the cytotoxicity of fiber posts and core composites has been recently investigated (26). However to date no study has evaluated the antibacterial properties of fiber posts.

In this study the antibacterial effects of a new silver fiber post have been evaluated, by using the agar disc diffusion test (ADT). The agar diffusion test has been widely used to test the antimicrobial activity of dental materials. A disadvantage of the ADT is that the results are highly influenced by the diffusibility of the material across the medium: the size of the inhibition zones does not indicate the absolute antimicrobial effect of the sealer. A material that diffuses more easily will probably provide larger zones of inhibition (27); other variables such as inoculum size, incubation time, and good material/agar contact may interfere with the results. If these variables are carefully controlled, reproducible results may be obtained (10,28). Moreover, direct contact between the fiber post and microorganisms may not be possible to achieve clinically. This is due by the layer formed between the post and the root canal walls by the resin cement used for cementation. However, it seems that AgNPs can diffuse in the surrounding resin cements to be effective against residual bacteria (29): for these reasons, ADT may be considered a valid method to assess the antimicrobial properties of the silver fiber post.

The cytotoxicity of the different fiber posts has been investigated, by using the MTT assay. The MTT test is a standard colorimetric assay for measuring the activity of enzymes that reduce the MTT to formazan (a salt blue) in the mitochondria, giving the substance a blue/purple color. This technique has been widely used to characterize the cytocompatibility of various dental materials (30).

The null hypothesis of this study was partially rejected. In fact the new silver fiber post demonstrated a fair antibacterial activity; while all the fiber posts tested did not present cytotoxic effects on human periodontal fibroblasts.

As concerns the antibacterial activity, the in vitro cytotoxicity of AgNPs on fungi, protozoa, gram-negative and gram-positive bacteria such as *Streptococcus mutans*, *Lactobacillus*, and *Staphylococcus aureus* has been recently demonstrated by several studies (31,32). Silver is antibacterial against periodontal pathogens and Streptococci in the oral cavity, but also prevents adhesion of bacteria to surfaces and formation of biofilms (33). Since microorganisms in biofilms are more resistant to antimicrobial agents than planktonic pathogens, the ability of AgNPs to penetrate through cell membranes readily is especially important (34). The antimicrobial mechanism of AgNPs remains unclear. It is possible that silver ions interact with the peptidoglycan cell wall, causing increased membrane permeability and, finally, cell death (35). Moreover, growth inhibition promoted by silver particles has been associated with the disruption of ATP production and DNA replication and with the generation of reaction oxygen species (ROS) (35).

In this study the new silver fiber post demonstrated alone antibacterial effects against all the three streptococcal strains tested. Nano fiber post with zirconia nanoparticles, was slightly cytotoxic on *S. mutans* and significantly lesser on *S. sanguis*. The other posts tested did not present antibacterial effects. Our results confirmed the antimicrobial effects of AgNPs. The incorporation of silver can give the fiber post a useful antibacterial activity, thus decreasing the development of recurrent caries and increasing the longevity of tooth restorations. Although no study has yet assessed the antibacterial activity of a silver fiber post; similarly to our results, many Authors have reported the antimicrobial effects of AgNPs incorporated in different dental materials. Some researchers have introduced nanosilver-gutta-percha, as an attempt to improve the antibacterial effect of gutta-percha (36). Other studies demonstrated the bactericidal activity of composite resins containing silver nanoparticles and the positive effects of AgNPs on MTA antimicrobial activities (32,37).

As regards biocompatibility, all the fiber posts tested reported a percentage of cell viability greater than 90%. All the posts tested did not present cytotoxic behavior and they can be considered biocompatible. These results fully embody the excellent biological safety performance of the fiber posts tested. Our findings also demonstrated the biocompatibility of the new silver fiber post, thus confirming the cytocompatibility of the incorporated AgNPs. These considerations are in accordance with previous studies, which reported the complete biocompatibility of silver nanoparticles. In a research by Shantiaee *et al.* the cytotoxicity of nanosilver-coated gutta-percha was found to be similar to the one of normal gutta-percha (38). Acosta-Torres showed that PMMA-AgNP compound for dental prostheses does not present cytotoxity or genotoxicity (39). Finally as concerns the incorporation of silver in restorative materials, it has been shown that AgNPs addition did not affect the cytotoxicity of the material (40).

## Conclusions

In this study, the antimicrobial effect of AgNPs incorporation into a new fiber post was investigated. The new silver fiber post reported an inhibitory effect against *S. mutans*, *S. salivarius*, *S. sanguis*. This antibacterial activity could decrease the occurrence of secondary caries, enhancing the longevity of the tooth-restoration complex. On the other hand all the fiber posts tested (including the post with incorporated AgNPs) proved to be biocompatible, suggesting that their application does not represent a threat to human health. However, more studies are necessary to determine the optimal concentration of the silver compound, in order to guarantee a fair antimicrobial effect without increasing its cytotoxicity.
